# Orf virus interferes with MHC class I surface expression by targeting vesicular transport and Golgi

**DOI:** 10.1186/1746-6148-8-114

**Published:** 2012-07-18

**Authors:** Frederic Emschermann, Michael R Knittler, Hanns-Joachim Rziha

**Affiliations:** 1 Present address: Department of Immunology, Interfaculty Institute for Cell Biology, University of Tuebingen, Auf der Morgenstelle 15, 72076, Tuebingen, Germany; 2 Friedrich-Loeffler-Institute, Federal Research Institute of Animal Health, Institute of Immunology, Greifswald-Insel Riems, Germany

**Keywords:** Orf virus, Parapoxvirus, MHC class I, Subversion, Immunomodulation, Golgi apparatus

## Abstract

**Background:**

The Orf virus (ORFV), a zoonotic Parapoxvirus, causes pustular skin lesions in small ruminants (goat and sheep). Intriguingly, ORFV can repeatedly infect its host, despite the induction of a specific immunity. These immune modulating and immune evading properties are still unexplained.

**Results:**

Here, we describe that ORFV infection of permissive cells impairs the intracellular transport of MHC class I molecules (MHC I) as a result of structural disruption and fragmentation of the Golgi apparatus. Depending on the duration of infection, we observed a pronounced co-localization of MHC I and COP-I vesicular structures as well as a reduction of MHC I surface expression of up to 50%. These subversion processes are associated with early ORFV gene expression and are accompanied by disturbed carbohydrate trimming of post-ER MHC I. The MHC I population remaining on the cell surface shows an extended half-life, an effect that might be partially controlled also by late ORFV genes.

**Conclusions:**

The presented data demonstrate that ORFV down-regulates MHC I surface expression in infected cells by targeting the late vesicular export machinery and the structure and function of the Golgi apparatus, which might aid to escape cellular immune recognition.

## Background

The Orf virus (ORFV; Parapoxvirus ovis) is the type species of the *Genus Parapoxvirus* belonging to the family *Poxviridae*. It is a skin epitheliotropic double-stranded DNA virus that causes pustular skin lesions in sheep and goats, known as contagious ecthyma [1]. Most interestingly, animals are not protected against ORFV re-infections, which might also be due to the short-lived ORFV-specific adaptive immunity. Orf is a zoonotic disease [2] that can be transmitted to humans by contact with infected animals. While Orf is usually a benign self-limiting illness, it can be very progressive in immune-compromised hosts [2].

Poxviruses provide considerable inventories of gene products that allow them to evade the host immune response [3]. It has been previously shown that ORFV encodes immunomodulators like ORFV IL-10, the GM-CSF- and IL-2-inhibitory factor (GIF) or the ORFV chemokine binding protein CBP, which have the ability to inhibit cytokine synthesis of monocytes [4-8]. These evasion strategies seem to play an important role in supporting ORFV replication and enabling repeated re-infections.

Cell-mediated immunity is critical for the clearance of virus-containing cells. Infected hosts normally react by activating their MHC I - mediated cellular immune response [9]. MHC I transmembrane glycoproteins function by binding intracellularly processed peptide antigens and presenting them on the cell surface to cytotoxic T cells [10]. During viral infection, a spectrum of antigenic peptides is displayed by MHC I molecules, resulting in the specific recognition of the infected cells by cytotoxic T cells (CTL). However, many viruses, including poxviruses [3,11], evade the T cell-mediated immune response, primarily by decreasing the levels of surface MHC I, thus reducing the presentation of pathogen-derived antigens [12] to escape cellular immunosurveillance mechanisms [13]. MHC I down-regulation of infected cells increases susceptibility to natural killer (NK) cells, and many viruses have also evolved strategies to escape this immune detection [14].

The ability to inhibit proinflammatory cytokines (TNF and IFN) that regulate MHC expression is a mechanism of poxviruses to prevent the up-regulation of MHC I [3]. The gene product M153R of myxoma virus interferes directly with the antigen presentation pathway and induces the loss of β2-microglobulin associated MHC I, both at the cell surface and in an intracellular post-Golgi compartment [15]. Genes of cowpox virus modulate the MHC I antigen processing and expression. The CPXV203 protein is responsible for decreased surface expression of mouse and human MHC I molecules by using the physiologic KDEL-pathway to retain MHC I in the ER [16,17], whereas the CPVX12 protein prevents TAP-dependent peptide loading [18,19].

We are interested to identify possible immune evasion mechanisms of ORFV, the type species of Parapoxvirus. Also *in vitro* propagation of wild-type ORFV is very restricted and mostly primary ovine or bovine cells are used, which limits the availability of MHC I or cell compartment specific reagents. Therefore, we took advantage from the Vero cell-adapted ORFV strain D1701-V to analyse virus induced alterations of MHC I surface expression in infected permissive Vero cells. We show that this Parapoxvirus impairs MHC I surface expression by structurally disrupting the Golgi apparatus. Most interestingly, Golgi fragmentation is accompanied by a defective intracellular MHC I transport, pronounced co-localization of MHC I and COP-I-vesicles, disturbed carbohydrate trimming of Golgi-localized MHC I molecules and a reduction of MHC I surface expression of up to 50%. In addition to these effects, we also noticed that the half-life of the remaining MHC I surface population is remarkably increased. All observed evasion phenotypes except for the MHC I half-life effect are linked to the expression of early ORFV genes. On the basis of our findings we postulate that ORFV modulates MHC I surface expression in infected cells by targeting the vesicular transport machinery and the structure and function of the Golgi apparatus. Thus, it is tempting to speculate that the discovered ORFV-mediated effects on MHC I act in concert to facilitate infection and allow the acute virus to replicate and shed prior to clearance by the host immune response.

## Results

### ORFV induces down-regulation of surface MHC I molecules

**Figure 1:**
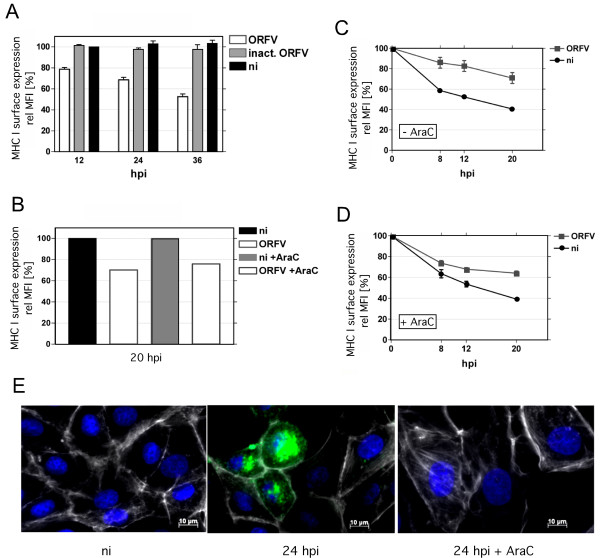
**Modulation of MHC I surface expression in ORFV-infected cells. (A)** Vero cells were harvested at 12, 24, and 36 hpi (m.o.i. 1.0) and stained with the anti-MHC I mAb W6/32 as described in Methods. The effect of non-replicating ORFV was tested by the use of ß-propiolactone inactivated ORFV (inact. ORFV; m.o.i. 1.0 before inactivation), non-infected (ni) cells were used as negative controls. The average of three separate virus culturing experiments is shown. ORFV infection decreased cell surface expressed MHC I. **(B)** Twenty hours post infection (m.o.i. 1.0), MHC I cell surface expression (W6/32) was determined by FACS in the presence and absence of AraC. No effect of AraC treatment on MHC I surface expression was observed. One representative experiment is shown. **(C)** ORFV infected (m.o.i. 1.0) or non-infected Vero cells were treated with BFA or **(D)** with BFA plus AraC. Virus infection increased the half-life of MHC I on the cell surface, determined at 8, 12 and 20 hpi using W6/32 anti-MHC I antibody by flow cytometry. The average of three independent experiments is shown in C, D. The relative mean fluorescence intensity (rel MFI) is given in percentages. **(E)** Infection (m.o.i. 1.0) of Vero cells (green staining) and the effect of AraC was controlled (24 hpi) by immunofluorescence studies using the mAb 13 C10 (diluted 1:1000) recognizing the late major envelope protein of ORFV. Nuclei and F-actin are stained blue by DAPI and white by phalloidin-TRITC, respectively.

Surface expression of MHC I was investigated in ORFV-infected and non-infected Vero cells by flow cytometry using the MHC I specific monoclonal antibody (mAb) W6/32 as described in *Methods*. As shown in Figure 1a, ORFV infection resulted in a significant decrease of the MHC I surface expression. Twelve hours post infection (hpi) about 80% of MHC I was detectable on the cell surface compared to non-infected cells, which was further reduced to 70% at 24 hpi, and to almost 50% at 36 hpi. These decreases were statistically highly significant as determined by One-way ANOVA analysis (P < 0.001). Reduction of MHC I surface expression was dependent on live, replicating ORFV. Thus, infection of the cells with β-propiolactone-inactivated virus did not change the amount of MHC I expressed on the surface of Vero cells (Figure 1a, inact. ORFV).

To analyze whether expression of early or late ORFV genes might be responsible for the MHC I down-regulation, AraC was used to inhibit viral DNA synthesis and thereby preventing intermediate and late gene expression of ORFV [6]. Figure 1b demonstrates that blocking of ORFV intermediate and late gene transcription (+ AraC) did not abolish MHC I down-regulation or affect MHC I surface presentation in non-infected cells. Infection of Vero cells and the effect of AraC were controlled by immunofluorescence studies using the mAb 13 C10, which is directed against the late major envelope protein of ORFV (Figure 1e).

### ORFV infection increases the half-life of remaining surface MHC I molecules

Virus-infected cells were treated with Brefeldin A (BFA) to examine the biological stability of cell surface expressed MHC I molecules. BFA prevents the anterograde MHC I transport from the endoplasmic reticulum (ER) to the Golgi apparatus, and thereby inhibits the supply of newly synthesized MHC I to the cell surface. This experimental design allows the analysis of the half-life of surface expressed pre-existing MHC I by using flow cytometry. BFA-treated, non-infected Vero cells showed a 40 and 60% reduction of surface MHC I after 8 and 20 h incubation, respectively (Figure 1c, ni). In contrast, virus-infected Vero cells showed at the same BFA-incubation time points only a marginal MHC I decrease of 10% and 30% (Figure 1c, ORFV). These results suggest that ORFV infection increases the half-life of the remaining MHC I surface population by affecting surface stability and/or recycling of MHC I molecules. To examine whether early and/or late ORFV gene expression might be responsible for the increase in MHC I surface survival, cells were additionally treated with AraC during ORFV infection and BFA treatment. Figures 1c, d show that the MHC I half-life on the surface of non-infected cells was not altered by AraC. In infected cells the presence of AraC has some neutralizing influence on the ORFV mediated half-life effect on surface MHC I (compare Figure 1c, d). Thus, the ORFV-dependent increase of MHC I surface stability might be partially controlled also by late gene products.

### ORFV infection does not influence MHC I-transcription

**Figure 2:**
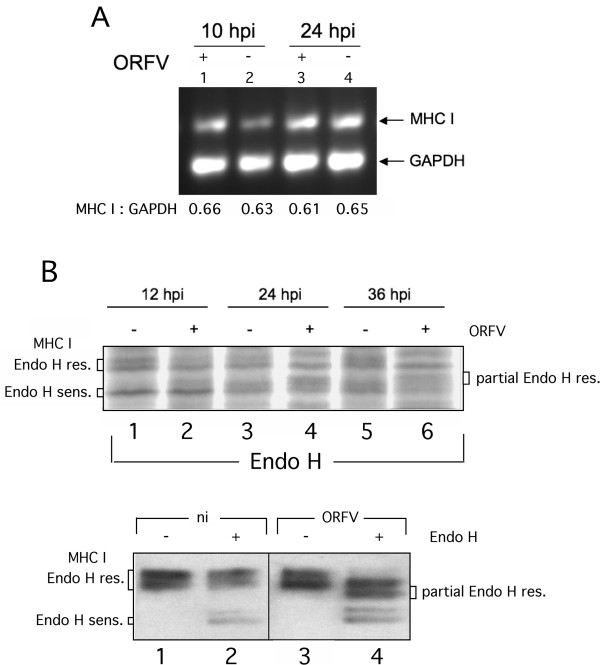
**Effects of ORFV-infection on expression and intracellular transport of MHC I . (A)** MHC I- and GAPDH-specific RT-PCR was performed as described in Methods. After gel electrophoresis the amplicon band intensities were quantified by densitometry and their calculated ratios are indicated below each gel lane. The transcription rate of MHC I was not affected significantly by ORFV infection. **(B, upper panel)** ORFV infection affects carbohydrate trimming of MHC I. Infected (+; m.o.i. 2.0) or not infected (−) cells were labelled with Trans-^35^ S-Label, lysed at 12, 24 and 36 hpi, and MHC I was immunoprecipitated with W6/32 antibody. The immunoprecipitates were digested with Endo H before separation by SDS-PAGE. Fluorographs were analyzed using GelEval 1.32 software (FrogDance Software). Endo H-resistant, -sensitive and partially Endo H-resistant MHC I forms are indicated. (**B, lower panel)** Infected (+; m.o.i. 1.0) or not infected (−) cells were lysed at 12 hpi, digested with Endo H and analyzed by Western blots probed with anti-MHC I mAb LY5.1.

A semi-quantitative RT-PCR was used to determine whether ORFV infection might influence the MHC I mRNA synthesis and thereby reduces MHC I surface expression. The amount of mRNA specific for the housekeeping gene GAPDH was related to the amount of MHC I mRNA at different times after infection. Each PCR product taken at the linear phase of PCR amplification was analyzed by gel densitometry. As shown in Figure 2a, the ratio of MHC I to GAPDH mRNA in non-infected cells ranged between 0.63 and 0.65 (Lanes 2 and 4), which remained almost unaltered 10 or 24 h after ORFV infection (lanes 1 and 3). Thus, the observed decrease of MHC I surface expression cannot be attributed to a prevention or inhibition of MHC I mRNA transcription by ORFV.

### ORFV infection disturbs carbohydrate trimming and maturation of MHC I

Next we analyzed whether and to what extent intracellular maturation of MHC I along the secretory route might be affected by ORFV infection. Endoglycosidase H (Endo H) – cleavage experiments were performed with anti-MHC I immunoprecipitates from detergent extracts of biosynthetically labelled, infected or non-infected Vero cells. Endo H is used to monitor posttranslational modification of glycosylated proteins within the Golgi. The MHC I-attached high mannose oligosaccharides are modified by a series of different ER and Golgi enzymes. Endo H is able to cleave oligosaccharides until the medial Golgi enzyme α-mannosidase II removes two mannose subunits. Since all later carbohydrate structures are Endo H-resistant, the enzyme monitors MHC I maturation within the late secretory route.

As can be seen from the SDS-PAGE analysis in Figure 2b upper panel, 12 h after ORFV infection intracellular MHC I-maturation is comparable in infected and non-infected Vero cells. In both situations we observed an approximately 1:1 signal ratio between Endo H-sensitive and -resistant MHC I molecules (Figure 2b upper panel, compare lanes 1 and 2). An additional minor species (approximately 10% of total MHC I signal) of partially resistant MHC I was also visible in infected cells (Figure 2b, upper panel, lane 2). After 24 and 36 h of infection, the population of Endo H-resistant MHC I was almost unaffected whereas the amount of Endo H-sensitive MHC I decreased by more than half (Figure 2b upper panel, lanes 4 and 6) as determined by densitometric scanning. Most importantly, the latter phenomenon was linked to a simultaneous increase of partially Endo H-resistant MHC I molecules by 45 and 55%, respectively. No such formation of unusual MHC I forms could be observed for non-infected control cells after 24 or 36 h of incubation (Figure 2b, upper panel, compare lanes 1, 3 and 5). The distinct behaviour of MHC I maturation in ORFV-infected cells was also seen in Western blot experiments, in which lysates of infected and non-infected Vero cells were assayed by using a different anti-MHC I antibody (mouse mAb, clone LY5.1, see Figure 2b, lower panel) with apparently higher specificity for the mature forms of MHC I. The two Endo H-resistant and -sensitive protein bands found after immunoprecipitation (Figure 2b, upper panel) or in Western blotting (Figure 2b, lower panel) by the two different anti-MHC I antibodies (W6/32 and LY5.1) most likely represent different allelic MHC I products expressed in Vero cells. Taken together, these findings suggest that ORFV-infection interferes with the functional requirements for proper carbohydrate trimming of MHC I within the *cis*- and/or *medial*-Golgi or the transport between the exocytic compartments.

### ORFV infection results in morphological changes of the Golgi apparatus

**Figure 3:**
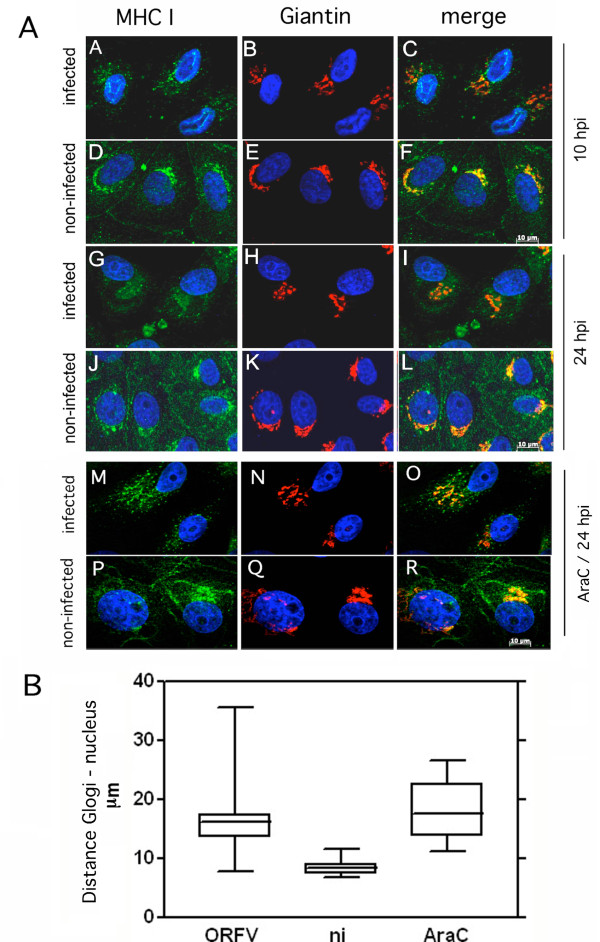
**ORFV-induced dispersion of the Golgi apparatus. (A)** Vero cells were infected (m.o.i. 0.5) and stained with the MHC I-specific mAb W6/32 (green) and the Giantin antibody specific for Golgi (red) after 10 hpi **(panels A-F)** and 24 hpi **(panels G-L)** or 24 hpi in the presence of AraC **(panels M-R)**. In infected cells MHC I dispersed into the cytoplasm and Golgi structures dispersed into the cytoplasm. Nuclei are stained blue by DAPI. Representative results of five independent experiments are shown. **(B)** The distances of the Golgi from the nucleus in non-infected and infected cells in the presence or absence of AraC were quantified using with AxioVision Rel. 4.8 software. The evaluation of 46 infected cells (ORFV), 20 infected and AraC-treated cells (ORFV/AraC), and 42 non-infected cells (ni) demonstrates significantly (*T* test: P < 0.0001) increased distance between Golgi and cell nucleus in infected cells. Box plots with median percentile were accomplished with GraphPad Prism 5 software.

Next, we investigated whether ORFV infection might affect the secretory pathway and Golgi transport of MHC I and thereby prevents intracellular trafficking of newly synthesized MHC I to the cell surface. Therefore, we analyzed infected cells by confocal immunofluorescence after co-staining of intracellular MHC I and Giantin, a main component of the *cis*- and *medial*-Golgi. The results in Figure 3a demonstrate that virus infection caused substantial changes in the localization patterns of MHC I and Giantin. Already 10 hpi, MHC I dispersed into the cytoplasm with a punctuated vesicular structure (Figure 3a - panel A) continuing to 24 hpi (Figure 3a - panel G), whereas MHC I in non-infected Vero cells showed a dense and ring-shaped perinuclear staining (Figure. 3a - panels D and J).

In non-infected cells, Giantin-staining was characterized by a compact perinuclear pattern (Figure 3a - panels E and K) that disappeared during ORFV infection and scattered throughout the cytoplasm (Figure 3a - panels B and H). Simultaneously, co-localization between Giantin and MHC I, which was clearly seen in non-infected cells (Figure 3a - panels F and L), was reduced during virus infection (Figure 3a – panels C and I) as verified by calculating the coefficient of co-localization (Pearson value; data not shown). The ORFV-induced Golgi spreading was also found in AraC-treated infected cells (Figure 3a, panels M to R) indicating the involvement of early ORFV gene(s). The ORFV-induced dislodgment of Golgi from its original nucleus-associated location into the cytoplasm could be confirmed by quantitative analysis of the distances between Golgi and nucleus in infected and non-infected cells (Figure 3b). The distance from the centre of the nucleus of each cell to the peripheral fringe of the Golgi was almost duplicated in infected cells, in the presence as well as in the absence of AraC, when compared to non-infected cells, and was highly significant according to *T* test (P < 0.0001).

**Figure 4:**
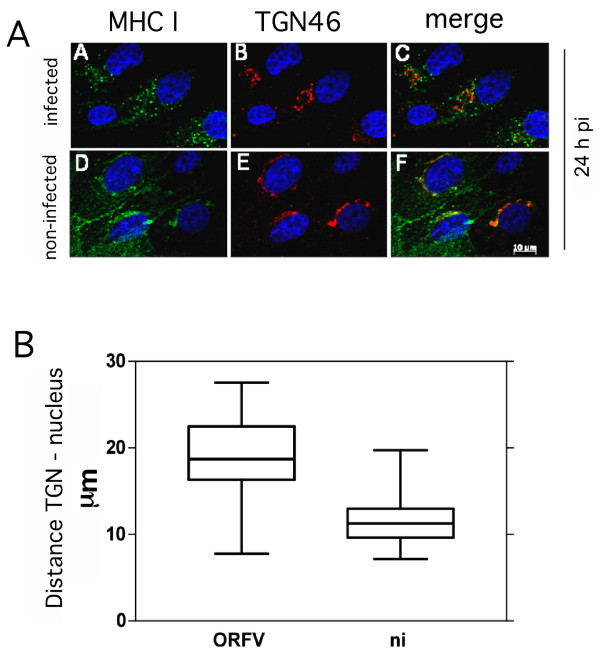
**Structural changes of the trans-Golgi network (TGN) after ORFV infection. (A)** Infected cells (m.o.i. 0.5) or non-infected cells were fixed 24 hpi and stained with W6/32 antibody (green) and anti-TGN46 antibody (red) **(panels A-F)**. Partial co-localization of MHC I and TGN can be seen in infected cells by merging the fluorescent images (merge). Nuclei are stained blue by DAPI. After infection TGN lost its perinuclear location and moved into the cytoplasm. A representative result of confocal fluorescence microscopy of three experiments is shown. **(B)** TGN-dislocation in ORFV infected cells. The distances of the TGN and the nucleus in infected and non-infected cells were quantified using with AxioVision Rel. 4.8 software (Zeiss). The evaluation of 27 infected and 27 non-infected cells is summarized as box plots and demonstrates an increased distance between TGN and cell nucleus in infected cells.

The *trans*-Golgi network (TGN) represents another important constituent of the late secretory route involved in exo- as well as endocytic processes [20]. The possible influence of ORFV on the TGN structure was examined with a TGN46-specific antibody. Partial co-localization between TGN46 and MHC I was visible in infected and non-infected cells. Similar to Giantin and MHC I, TGN46 lost its prominent perinuclear distribution after virus infection in favour of a punctuated vesicular pattern within the cytoplasm (Figure 4a, compare panels A and D, B and E), which was also seen in infected cells arrested for early gene expression by AraC (data not shown). Quantitative analysis of the images (Figure 4b) revealed a significantly increased distance (P < 0.0001) between TGN and nucleus (17 to 23 μm) in comparison to non-infected cells (9 to 12 μm). In summary, in virus infected cells Golgi and TGN are structurally dispersed into the cytoplasm and these processes are linked to early gene expression.

### Influence of ORFV on the intracellular transport of MHC I molecules

**Figure 5:**
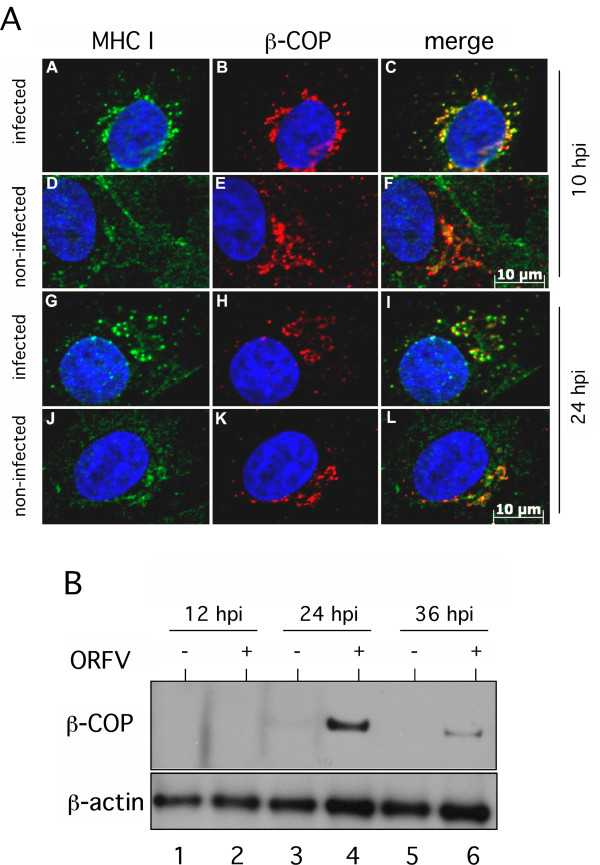
**ORFV-infection interferes with COP-I mediated vesicular transport. (A)** MHC I co-localizes with COP-I vesicles after ORFV infection. In infected cells (m.o.i. 0.5) MHC I was stained with mAb W6/32 (green) and anti-β-COP antibody (red). A representative result of three experiments at 10 hpi **(panels A-F)** and 24 hpi **(panels G-L)** is shown. Cell nuclei are stained blue with DAPI. A distinct co-localization (merge, yellow) of MHC I and β-COP was found in infected cells. Note that non-infected cells had to be photographed with longer exposure times as infected cells for the sake of better MHC-I/ß-COP visualization. **(B)** ORFV induced expression levels of β-COP. ß-COP (95 kDa) was detectable by Western blot analysis in infected cells (m.o.i. 1.0; lanes +) during 24 to 36 hpi. Detection of cellular ß-actin demonstrates comparable protein loading.

Since ORFV-infection leads to a fragmentation of Golgi, we explored the viral influence on Golgi-transport of MHC I. COP-I is a protein complex that coats vesicles transporting polypeptides between different Golgi compartments and from the *cis*-Golgi back to the ER [21]. Therefore, we analyzed intracellular staining of MHC I and COP-I-component β-COP in infected and non-infected cells by fluorescence microscopy. Non-infected Vero cells displayed a characteristic juxtanuclear staining pattern of MHC I (Figure 5a - panels D and J) but only partial intracellular co-labelling of MHC I and β-COP (Figure 5a - panels F and L). In infected Vero cells a prominent perinuclear and vesicular MHC I-staining was observed 10 hpi that, however, dispersed into the cytoplasm after 24 hpi (Figure 5a - panels A and G). In contrast to non-infected cells, MHC I/β-COP co-localization could be seen for both infection time points (Figure 5a - panels C and I) confirmed by Pearson value calculation (data not shown). It must be noted that the non-infected cells were photographed with longer exposure time for the sake of better visualization. Additional AraC experiments showed that this effect is also controlled by early ORFV gene expression (data not shown).

Given expression levels of β-COP were analyzed by Western blot experiments in infected and non-infected cells. Figure 5b demonstrates that the 95 kDa β-COP protein was hardly detectable in cell extracts of non-infected Vero cells, most likely due to the fact that β-COP, like other COP-I components, does not stably exist out of the coatomer complex [22]. Nevertheless, 24 h after ORFV infection β-COP was clearly visible with reduced amounts expressed after 36 hpi suggesting that the population of stably assembled COP-I structures is drastically enlarged in infected cells. Comparable protein loading was controlled by β-actin staining (Figure 5b, lower panel). Taken together, our findings provide evidence that the amount of MHC I-containing stable COP-I vesicles increased significantly during the first 24 hours after ORFV infection.

## Discussion

The presented study shows that cellular ORFV infection leads to structural dispersion of the Golgi/TGN compartments and enrichment of COP-I vesicular structures. These processes are accompanied by an increase in the steady state expression of β-COP (Figure 5b), defective carbohydrate trimming of MHC I within the Golgi (Figure 2b), reduction of surface expressed MHC I molecules and a prolonged half-life of pre-existing MHC I on the plasma membrane (Figure 1). Upcoming studies have to prove whether the described interferences of ORFV with the MHC I expression also occur in natural host cells.

Our findings demonstrate that in ORFV-infected cells the intra-Golgi- and endosome/TGN-transport of MHC I was severely disturbed. ORFV seems to utilize early gene expression to block MHC I export within the late secretory route and thereby reduces MHC I surface expression. As shown by our experiments ORFV alters the perinuclear localization as well as the overall structure of the Golgi and TGN in infected Vero cells. Similar effects on the Golgi have also been described for a variety of different viruses. Early gene expression of Varicella zoster virus leads to MHC I down-regulation by impairing its transport to the cell surface [23]. A late event in the reproductive cycle of Herpes simplex virus type 1 causes fragmentation and dispersal of the Golgi in infected Vero cells, which coincides with virion assembly [24]. The infection with human rhinovirus 1A (HRV-1A) induces Golgi-fragmentation into vesicles that appear to be used as a substrate for viral RNA replication [25]. Another positive-strand RNA virus, the poliovirus, induces dramatic disruption of the Golgi with consequences for the secretory complex [26,27]. Furthermore, it is known that vaccinia virus becomes enwrapped by cisternae derived from the intermediate compartment between ER and Golgi stacks as well as the TGN [28]. Recently Tan et al. also observed fragmentation of the Golgi during ORFV infection, and reported the Golgi localization of an ORFV envelope protein during late stage of infection [29]. The authors suggested that it is concealed between two Golgi membranes, which are forming wrapped mature virions. In the present study, the destruction of the Golgi structure is clearly not linked to virus envelope formation since the observed structural modifications are also visible in the presence of AraC, which prevents the expression of late ORFV genes essentially required for the virus envelope.

ORFV-infected cells are characterized by a reduced amount of newly synthesized MHC I on the plasma membrane as well as a prolonged half-life of the remaining pre-existing surface MHC I molecules (Figure 1). Down-regulation of MHC I is clearly AraC-insensitive and thus apparently linked to the expression of early ORFV genes whereas it cannot be excluded that the observed MHC I half-life effect might be also controlled by late ORFV gene expression. It is tempting to speculate that the respective viral gene products target compartments within the late secretory route. Since structural and functional integrity of the TGN are essentially required for endosomal/TGN-trafficking, the observed disruption of the TGN in infected cells (Figure 4) might be suspected to interfere with endocytosis as well as endosomal recycling of MHC I. A similar phenotype has been described for the HPV16 protein E5 [30], which mediates disruption of the exo- and endocytic trafficking, including transport of the MHC I [30], which causes reduced MHC I surface presentation and extends the half-life of the remaining MHC I molecules on the plasma membrane (M. R. Knittler, manuscript in preparation).

The ORFV infection leads to an accumulation of MHC I in COP-I vesicles (Figure 5a). COP-I is the cytoplasmic membrane-coat complex (coatomer) of seven distinct proteins and is required for both anterograde and retrograde transport in the secretory pathway [31,32]. The observation that ORFV infection increases the cellular expression levels of β-COP (Figure 5b) and the amount of COP-I vesicular structures suggests inhibition of uncoating of COP-I vesicles by ORFV. The identification of responsible ORFV protein(s), as found in Coxsackievirus [33], requires further detailed studies. In contrast to vaccinia virus, which hijacks the COP-I coatomer for viral particle formation [34], no correlation between accumulation of COP-I vesicles and viral biogenesis was observed, since the ORFV-mediated effect was also detectable in the presence of AraC.

The Endo H-experiments suggest that destruction of Golgi and TGN structures as well as intracellular accumulation of MHC I in COP-I vesicles is accompanied by impaired post-ER maturation of the N-linked carbohydrates of MHC I. In contrast to non-infected cells, a substantial amount of the MHC I molecules exhibits partial Endo H-resistance in ORFV-infected cells indicating that these molecules are not correctly processed by carbohydrate-trimming within Golgi. This reminds of the defective maturation of MHC I in the presence Concanamycin B, a specific inhibitor of the vacuolar type H(+)-ATPase [35], suggesting that ORFV infection not only affects the intracellular location and structure of Golgi and TGN, but also the functional pH conditions within these two compartments.

In addition to MHC I, ORFV infection also interferes with the surface expression of the transferrin receptor (TfR, CD71) (data not shown), which suggests that the ORFV-induced reduction of MHC I-antigen presentation is mediated by subversion of the host cell export machinery and not via specific targeting of MHC I molecules*.* Thus, one could assume that the ORFV-mediated modulation of vesicular transport has a more pleiotropic effect that also includes the reduction of antigen presentation and thereby provides an immune subversion strategy in advantage of the viral pathogen.

ORFV does clearly not interfere with the expression of MHC I molecules (Figure 2a) but uses an evasion strategy that accumulates newly synthesized MHC I molecules within the late secretory pathway (COP-I vesicles) possibly to down-modulate MHC I presenting viral antigens (for evasion of a cytotoxic T cell -mediated response), while simultaneously increasing the half-lives of pre-existing self peptide MHC I complexes at the plasma membrane (for evasion of an NK cell-mediated response). This suggests that ORFV like other large DNA viruses (e.g. Herpesviruses) uses different evasion strategies to interfere with antigen presentation at different levels of MHC I processing.

## Conclusion

We assume that the reduction of surface expressed MHC I and the impaired structure and function of the Golgi apparatus, which are possibly controlled by different ORFV gene products, independently affect intracellular transport and surface stability of MHC I and cooperatively undermine immune recognition of ORFV-infected cells by CTLs as well as NK cells. In view of the fact that the immunity elicited by ORFV is short-lived, and animals can be repeatedly infected [2], MHC I subversion may contribute to rescuing ORFV from host immunity and supporting viral replication in epidermal cells.

## Methods

### Cells and virus

The attenuated ORFV strain D1701-V was propagated and titrated in Vero cells as described [36]. Virus inactivation was achieved with 0.05% (v/v) β-Propiolactone (Serva) by incubation at 37 °C for 4 h and maintaining the pH-value of 7.6. After overnight incubation at 4 °C the supernatant was collected by centrifugation and plaque assays proved the successful virus inactivation.

### Antibodies

The mouse mAb W6/32 specific for HLA-ABC also recognizing simian MHC I [37] was used for flow cytometry, confocal fluorescence microscopy and immunoprecipitation. LY5.1 is a mAb recognizing MHC class I heavy chains of HLA-ABC (Acris). Antibodies specific for Giantin, TGN46 and β-COP were purchased from Abcam, the antibody against ß-actin from Sigma-Aldrich. The mAb 13 C10 is directed against the 39 K major envelope protein of ORFV [38] and was a generous gift of C. McInnes and P. Nettleton (MRI, Pentlands Science Park, Penicuik, Scotland). As second antibodies we used anti-mouse FITC-conjugated antibody (Dianova), anti-mouse Alexa Fluor 488- and Alexa Fluor 555-conjugated antibodies and anti-rabbit Alexa Fluor 488- and Alexa Fluor 555-conjugated antibodies (Fisher Scientific, Invitrogen) and HRP-conjugated anti-rabbit IgG (Dianova).

### Flow cytometry

Vero cells were infected with a m.o.i. of 1.0, harvested and stained successively with primary antibody and FITC-conjugated secondary antibody for 30 minutes at 4 °C. Brefeldin A (BFA, Sigma-Aldrich) was used in a concentration of 10 μg ml^-1^, cytosine arabinoside (AraC, Sigma-Aldrich) was added (40 μg ml^-1^) during virus infection. For viable cell determination dead cells were stained with 7-AAD (BD Bioscience) prior to FACS analyses using a FACSCalibur (BD Bioscience) and CellQuest Pro (BD Bioscience).

### RNA isolation and semi-quantitative reverse transcription PCR

RNA kit (SurePrep True Total RNA Purification Kit, Fisher Scientific) was used to isolate total RNA from infected (m.o.i. 1.0) and non-infected Vero cells according to the manufacturer's instructions. RNA was treated with DNase (DNA-free, Ambion) and 300 ng were used for RT-PCR. Specific RNA of MHC I and Glyceraldehyde-3-phosphate dehydrogenase (GAPDH) as a housekeeping gene was amplified by RT-PCR according to the manufacturer's recommendation (OneStep RT-PCR Kit, Qiagen) in a total volume of 10 μl, using GAPDH-specific primers at an annealing temperature of 64 °C [39] or using MHC I generic primers at an annealing temperature of 62 °C [40]. PCR products were taken during the linear phase of amplification, separated by gel electrophoresis and the amplicon DNA band intensities were quantified using GelEval 1.32 software (FrogDance Software).

### Immunofluorescence

Vero cells were grown and infected (m.o.i. 0.5) in chamber slides (BD Biosciences) and fixed with 2% (v/v) methanol-free formaldehyde (Pierce, Fisher Scientific) in PBS and permeabilized with 0.2% (v/v) Triton-X100 (Sigma-Aldrich) in PBS. After 30 minutes blocking at room temperature in 5% (v/v) FCS in PBS, all antibody incubations were performed in PBS containing 1% (v/v) FCS for 30 minutes at 37 °C. F-actin was stained with Phalloidin-TRITC (Sigma-Aldrich), nuclei were stained with DAPI (1 μg ml^-1^, Sigma-Aldrich) before embedding of slides in Mowiol-DABCO. Confocal microscopy was performed with ApoTome confocal fluorescence microscope (Axiovert 200 M; Zeiss) and arranged with AxioVision Rel. 4.8 (Zeiss). The Pearson coefficient showing degree of colocalization was determined using the program CoLocalizer Express (CoLocalizer).

### Biosynthetic labelling and immunoprecipitation of proteins

Cells were starved for one hour in methionine-cysteine free Dulbecco’s modified Eagle’s medium (DMEM; Gibco) supplemented with 4 mM L-glutamine and 1 mM Na-Pyruvate, followed by incubation for additional 12 h in the presence of 10.5 mCi ml^-1^ Trans-^35^ S-Label (MP Biomedicals). Washed labelled cells were solubilised in PBS containing 1% Triton- X100 (Sigma-Aldrich) on ice for 45 minutes. After centrifugation at 14.000 rpm for 5 minutes the supernatants were used for immunoprecipitations at 4 °C overnight with anti MHC I mAb W6/32, which has been coupled directly to cyanogen bromide-activated sepharose (Amersham Life Sci.). Precipitates were digested with 10 mU of Endo H (Sigma-Aldrich) for 12 h at 37 °C and MHC I was eluted with 2.4 M urea, 2% SDS, 20% Glycerine, 125 mM Tris (pH 6.8) for 5 minutes at 95 °C prior to SDS-PAGE. Following electrophoresis fixed and dried gels were exposed to X-ray films (Kodak).

### SDS-PAGE and western blot analysis

Non-infected and infected (m.o.i. 1.0) cells were dissolved with 1% (v/v) Triton- X100 (Sigma-Aldrich) in PBS for 30 minutes at 4 °C. SDS-PAGE and Western Blot were performed as reported [41]. All antibodies were diluted in 1 x RotiBlock (Roth) and for enhanced chemiluminescence (ECL) the substrate Immobilon Western HRP (Millipore) was used. X-ray films for ECL were purchased from Pierce (Fisher Scientific).

### Statistical analysis

Statistical significances were evaluated by One-way ANOVA analysis (Figure 1) or by the *T* test (Figures 2 and 3) using GraphPad Prism 5 software (La Jolla).

## Competing interests

The authors declare that they have no competing interests.

## Authors’ contribution

JR carried out the studies, participated in the design of the studies and drafted the manuscript. FE participated in flow cytometry analysis and in the design of the studies. MRK and H-JR designed and coordinated the studies, aided in the interpretation of the data and drafted the manuscript. All authors read and approved the final manuscript.
